# Accuracy of two methods to detect the presence of halitosis: the volatile sulfur compounds concentration in the mouth air and the information from a close person

**DOI:** 10.1590/1678-7757-2022-0412

**Published:** 2023-05-01

**Authors:** Nádia Cristina Pinheiro RODRIGUES, Alexandre ABRÃO, Paulo NADANOVSKY

**Affiliations:** 1 Universidade do Estado do Rio de Janeiro Instituto de Medicina Social Hesio Cordeiro Rio de Janeiro RJ Brasil Universidade do Estado do Rio de Janeiro, Instituto de Medicina Social Hesio Cordeiro, Rio de Janeiro, RJ, Brasil.; 2 Escola Nacional de Saúde Pública Sérgio Arouca Fundação Oswaldo Cruz Rio de Janeiro RJ Brasil Escola Nacional de Saúde Pública Sérgio Arouca, Fundação Oswaldo Cruz, Rio de Janeiro, RJ, Brasil.; 3 Universidade do Estado do Rio de Janeiro Departamento de Gastroenterologia Rio de Janeiro RJ Brasil Universidade do Estado do Rio de Janeiro, Departamento de Gastroenterologia, Rio de Janeiro, RJ, Brasil.

**Keywords:** Halitosis, Mouth, Data accuracy, Reproducibility of results, Oral diagnosis, Epidemiology

## Abstract

This study aimed to analyze the accuracy of two methods for detecting halitosis, the organoleptic assessment by a trained professional (OA) with volatile sulfur compounds (VSC) measurement via Halimeter^®^ (Interscan Corporation) and information obtained from a close person (ICP).

**Methodolody:**

Participants were patients and companions who visited a university hospital over one year period to perform digestive endoscopy. A total of 138 participants were included in the VSC test, whose 115 were also included in the ICP test. ROC curves were constructed to establish the best VSC cut-off points.

**Results:**

The prevalence of halitosis was 12% (95%CI: 7% to 18%) and 9% (95%CI 3% to 14%) for the OA and ICP, respectively. At the cut-off point >80 parts per billion (ppb) VSC, the prevalence of halitosis was 18% (95%CI: 12% to 25%). At the cut-off point >65 ppb VSC, sensitivity and specificity were 94% and 76%, respectively. At the cut-off point >140 ppb, sensitivity was 47% and specificity 96%. For the ICP, sensitivity was 14% and specificity 92%.

**Conclusions:**

VSC presents high sensitivity at the cut-off point of >65 ppb and high specificity at the cut-off point of >140 ppb. ICP had high specificity, but low sensitivity. The OA can express either occasional or chronic bad breath, whereas the ICP can be a potential instrument to detect chronic halitosis.

## Introduction

How can halitosis be measured and diagnosed? How can people know whether or not they have bad breath? These and other inquiries have fueled several attempts to develop halitosis detector instruments in the last century.^1^

Halitosis is a universal symptom of important social impact, which occurs in the chronic and occasional form.^2 - 4^ It occasionally affects about 15% to 58% of the population and probably around 15% (95% CI 11% to 19%) of the population present bad breath constantly^5 - 7^ . Inconsistencies between estimates of halitosis prevalence probably result from the different methods and criteria used to define the presence of halitosis.^8 , 9^

Population survey data in Japan indicated that time of day is an important factor to consider in halitosis prevalence estimates. That survey detected a higher frequency of bad breath in the morning compared to the afternoon and also in individuals with more than 2.5 hours after the last oral activity (food intake, brushing, etc.).^10^

Halitosis can be measured by organoleptic measurements, portable clinical devices (eg. OralChroma, *Halimeter* ^®^) *,* gas chromatography analysis, and even by interview (eg. HALT questionnaire).^11^ The breath odor organoleptic assessment by a trained professional (OA) is considered the gold standard of halitosis diagnostic methods.^12 - 14^ However, due to concerns with its accuracy and reproducibility, influenced not only by the degree of aspiration (fast or long), but also by the interference from other factors (psychological, cultural, physiological, among others). Therefore, alternative methods that offer greater comparability are needed.^12^ In this context, more objective instrumental measures are desirable, although the accuracy of such diagnostic methods is still questionable.^12 , 15^

One portable breath meter is the *Halimeter* ^®^ (Interscan Corporation - https://www.halimeter.com/). This instrument accurately reflects the levels of hydrogen sulfide; however, other volatile sulfur compounds (VSC) detected by gas chromatography may be underestimated. The *Halimeter* ^®^ is not able to differentiate the types and concentrations of sulfuric components in breath samples and, in addition, it needs periodic recalibration. The reproducibility of this method has been considered satisfactory, especially for stationary VSC levels.^12 , 16^

Interviews with participants are infrequent in oral odor investigations, probably since halitosis self-assessment is a questionable method for bad breath diagnosis.^17 - 20^ Interview with a close person (ICP) to detect halitosis is rare in scientific literature. One study estimated that the prevalence of halitosis using ICP was around 15% (95%CI: 11% to 19%).^7^ This seems to be an interesting method as it overcomes the limitations of self-reporting of malodor whilst retaining the subjective measurement of malodor.^21^

Despite previous efforts, there is insufficient scientific evidence guiding clinicians to the different halitosis detection methods. This fact may reflect the multi-causality of this symptom concerning the numerous circumstances and clinical conditions associated with it.^22^ Instrumental measurements of certain VSCs are useful, but do not appear to be sensitive and specific enough to establish universally acceptable standards. Considering that few studies have estimated the accuracy of halitosis diagnostic methods and that halitosis information by the ICP is a strategy that has rarely been evaluated, this study aimed to analyze the accuracy of ICP and the VSCs concentration in mouth air for the detection of halitosis, compared to the OA of a trained professional.

## Methodology

### Participants and study site

This was a cross-sectional study, whose participants were patients who attended the university hospital of the *Universidade do Estado do Rio de Janeiro* from March 1, 2006, to February 28, 2007, to undergo digestive endoscopy, and their companions.

### Inclusion criteria

The inclusion criteria were: 1) Patients and companions who attended the digestive endoscopy service in the afternoon; 2) Age ≥18 years; 3) Not being a smoker; 4) No antibiotic treatment in the previous four weeks. Everyone who met these criteria was included in the VSC accuracy study. All patients included were fasting for twelve hours.

To participate in the ICP accuracy study, in addition to the previous four criteria, three more criteria were added: 1) Absence of olfactory disorder; 2) Living near the study site; 3) Availability for meeting three days every week.

**Figure 1:**
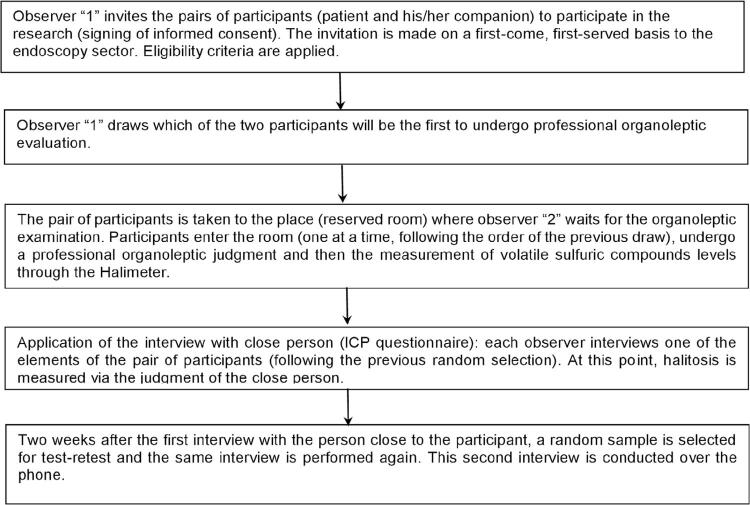
Phases of data collection

A total of 138 participants were eligible for the VSC study and, of this group, 115 people met all the inclusion criteria and were included in the two analyses, the VSC and the ICP. The stages of data collection are described in Figure 1 .

### Test-retest reliability

For the ICP test-retest reliability study, the participants were randomly selected and reinterviewed by phone, fifteen days after the first interview at the hospital. The selection process started in the second semester of the study period and consisted of drawing one week every month of data collection. All participants included in these randomly selected weeks also participated in the reliability study. The retest weeks were drawn by generating random numbers from the statistical software *OpenEpi* Version 3.01. Of the 53 participants selected for ICP test-retest reliability study, 13 were lost (25%). The losses that occurred were due to the incorrect telephone number information of the close person or to the impossibility of locating the person after three attempts at alternate times. In the end, 40 individuals were included in the reliability study.

### Definition of halitosis

For the gold standard, OA, the subject’s halitosis was measured by organoleptic assessment by a trained professional and was classified into one of the five following scores: 0- no halitosis or very good breath odor; 1- mild halitosis or good breath odor; 2- moderate halitosis or moderate breath odor; 3- severe halitosis or bad breath odor; 4- very severe halitosis or very bad breath odor. Two different cut-off points were established to define the presence of halitosis: a specific cut-off point, which considers scores 3 and 4 as the presence of halitosis; 2) a sensitive cut-off point, which considers scores 2, 3, and 4 as the presence of halitosis.

During the organoleptic examination, the participant was instructed to keep the mouth closed for one-minute breathing through the nose, and then count from one to ten, with the mouth at a distance of approximately 20 centimeters from the trained professional’s nose. To introduce blinding to the halitosis measurement, the OA of patients and companions were conducted without the trained professional knowing whether the participant was a patient or a companion. The results of the exams and surveys performed on companions are not reported in the present study.

For the ICP accuracy test, halitosis was classified by the same five scores as the gold standard (OA), and defined by the same specific and sensitive cut-off points.

For the VSC accuracy test, three cut-off points were used to define the presence of halitosis compared to the specific cut-off of OA: >65 VSC parts per billion (ppb), >80 VSC ppb, and >140 VSC ppb. Additionally, other three cut-off points were used to define the presence of halitosis compared to the sensitive cut-off of OA: >35 VSC ppb, >50 VSC ppb, and >80 VSC ppb.

### Random selection logistics

Observer “1” chooses which of the two participants will be the first to go through the OA evaluation.

The pair of participants is taken to the place (reserved room) where observer “2” waits for the beginning of the organoleptic measurement. The participants enter the room (one at a time, following the order of the previous draw), undergo a professional organoleptic assessment, and, then, *Halimeter* is used to measure VSC levels *.*

### ICP survey

**Figure 2:**
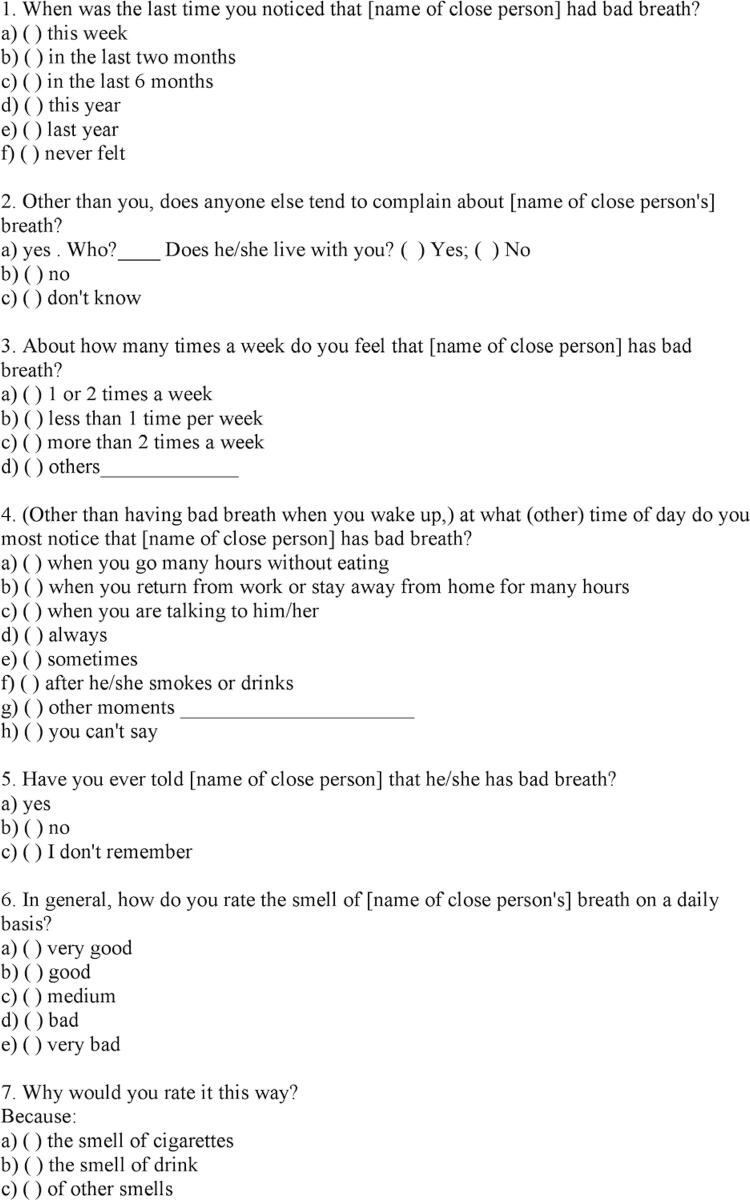
Questionnaire module for detection of halitosis via interview with a close person

After the OA and VSC data collection, the ICP data were obtained by face-to-face interviews with both members of the selected pairs (patients and their companions). The questionnaire module for halitosis detection by ICP consisted of seven closed or semi-open questions ( Figure 2 ), and a sixth question was included in this study: “In general, how do you evaluate the breath odor of (the patient’s name)?”.

Two weeks after the first ICP, a random sample of people who answered the first questionnaire was selected for test-retest and the same interview was conducted by telephone.

### Data analysis

The prevalence of halitosis and 95% confidence intervals (95%CI) were calculated and reported according to each measurement strategy, that is, OA, VSC, and ICP.

For the test-retest reliability analysis of the ICP, simple and weighted Kappa coefficients (quadratic weighting) were calculated.^23^

To define the VSC cut-off points, a receiver operation characteristic curve (ROC) was constructed, using the OA as a reference. To compare specific and sensitive cut-offs of OA, three cutoff points were chosen according to their sensitivity and specificity, that is, a point with high sensitivity, another with intermediate sensitivity and specificity, and a point with high specificity. The area under the curve and the 95%CI were calculated.

Frequency tables were constructed to report differences in the prevalence of halitosis among the gold standard (OA), VSC, and ICP. The McNemar test^24^ and the Kappa coefficient were used to reinforce the estimates of agreement among exams. Sensitivity, specificity, predictive values, and 95%CI were estimated for the VSC and the ICP assessments.

Graphic models were used to supplement the analysis. All analysis procedures were performed using the statistical software Stata 12.0 ( *StataCorp* ., *College Station, TX* ).

## Results

The estimated prevalence of halitosis for each measurement method was 12% (95%CI: 7% to 18%), 9% (95%CI: 3% to 14%), and 18% (95%CI: 12% to 25 %) for the OA (specific cutoff), ICP (specific cutoff), and VSC (>80 VSC ppb), respectively.

Table 1 shows the test-retest reliability of ICP. The greatest agreements were observed between the “good odor” scores. The greatest disagreements occurred between the “good odor” and “moderate odor” scores, that is, the individual’s breath was defined as “good odor” in the first interview but as “moderate odor” in the second interview. There was a high coefficient for a global agreement. The simple Kappa coefficient, calculated at the specific cut-off point, indicated excellent reproducibility (Kappa=0.79; 95%CI: 0.38 to 1). The weighted Kappa coefficient indicated moderate agreement between the scores (weighted Kappa=0.51 - p-v<0.001).

**Table 1:** Test-retest reproducibility of the presence of halitosis according to the interview with a close person (ICP); patients and companions who attended the digestive endoscopy service at one university hospital, Rio de Janeiro, Brazil

Agreement of scores		Information from a Close Person (ICP)					
two weeks apart		Very bad odor	Bad odor	Moderate odor	Good odor	Very good odor	Total
Retest Information from the same Close Person after two weeks	Very bad odor	**0**	0	0	0	0	0
	Bad odor	0	**2**	0	0	0	2
	Moderate odor	0	0	**8**	7	1	16
	Good odor	0	1	2	**14**	4	21
	Very good odor	0	0	0	0	**1**	1
	Total	0	3	10	21	6	40

n	Agreement	Kappa		Kappa		Kappa (w^2^)	
	Global Kappa (cut-off: score ≥ 3)	(2 cat; cut-off: score ≥ 2)		(2 cat; cut-off: score ≥ 3)		(5 cat)	

40	0,98	0,52		0,79		0,51	
		(CI 95% 0.30 – .74)		(CI 95% 0.38 – 1)		(p-value<0.001)	

cat = categories; w2 = quadratic weighting; halitosis cut-off points: score > 2 = very severe or severe versus moderate, mild or absent halitosis; score > 1 = very severe, severe, or moderate halitosis versus mild or absent

Test-retest - 2-week interval between interviews.

**Figure 3:**
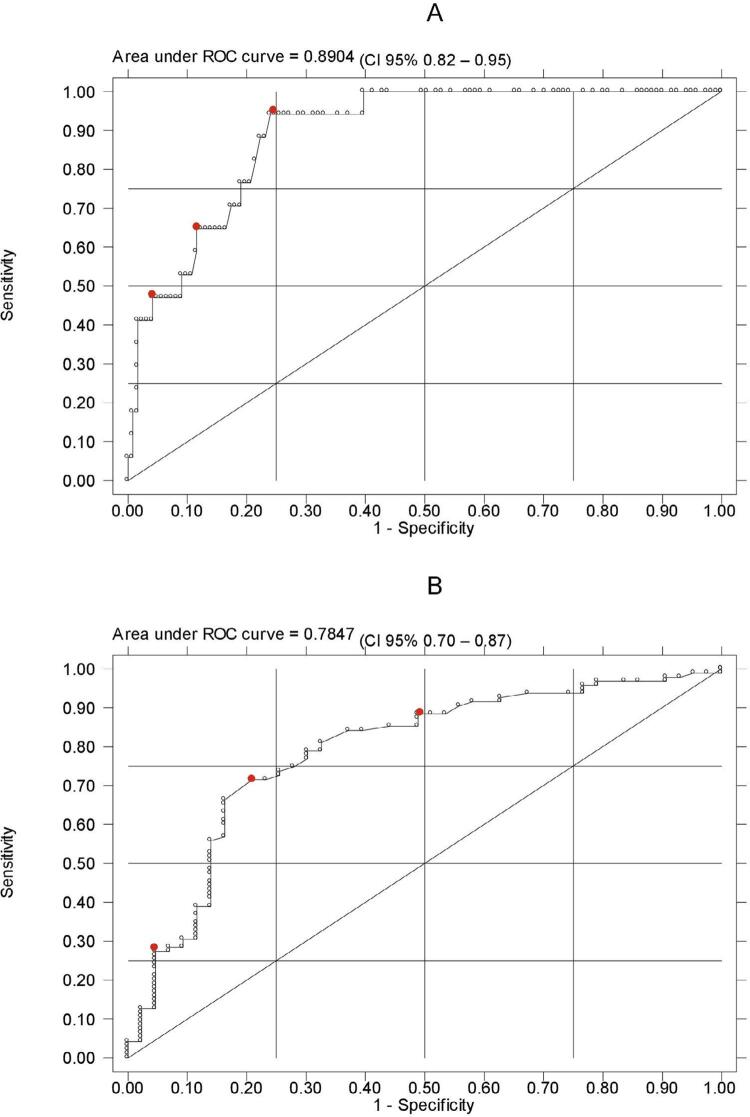
ROC curves of volatile sulfur compounds (VSC) in the classification of the presence of halitosis. A) Specific cut-off points of the organoleptic assessment (OA); B) Sensitive cut-off points of the OA ROC = Receiver Operating Characteristic. Gold Standard: organoleptic assessment by a trained professional (OA). Specific cut-off point (score>2) indicates very severe or severe halitosis; Sensitive cut-off point (score>1) indicates very severe, severe, or moderate halitosis. Red points represent the cut-off points analyzed in the measurement of VSC.

Figure 3 shows VSC ROC curves, considering the two definitions of halitosis based on the gold standard (OA): specific and sensitive cut-off points. The highlighted points represent the cutoff points investigated in this study. The greater area under the curve is observed in the specific cut-off (0.89; 95%CI: 0.82 to 0.95).

Table 2 presents the distribution of the VSC and ICP scores compared to the OA at the specific cut-off point. The predominance of true negative values (TN) is observed in all analyzed cut-off points. By increasing the test cut-off point, a percentual reduction of false positives is observed at the expense of an increase in the false negatives percentage.

**Table 2:** Distribution of volatile sulfur compounds (VSC) scores and information from a close person (ICP) compared to organoleptic judgment by a trained professional (OA) at the specific cut-off point (i.e. bad breath means very severe or severe halitosis)

Halitosis		Organoleptic Judgment (OJ) – specific cut-off		Total
		Yes	No	
Test:	Yes	16	29 (24%)^1^	45
VSC	No	1 (6%)^2^	92	93
Cut off = 65ppm	**Total**	17	121	138
Test:	Yes	11	14 (12%)	25
VSC	No	6 (35%)	107	113
Cut off = 80ppm	**Total**	17	121	138
Test:	Yes	8	5 (4%)	13
VSC	No	9 (53%)	116	125
Cut off = 140ppb	**Total**	17	121	138
	Yes	5	37 (41%)	42
Test:	No	9 (64%)	54	63
ICP Cut off sensitive	**Total**	14	91	105
Test:	Yes	2	7 (8%)	9
ICP Cut off specific	No	12 (86%)	84	96
	**Total**	14	91	105

Percentage of ^1^false positive and ^2^false negative.

Interview with a close person (ICP) scores cut-off points: 1) specific cut-off point (score > 2) = very severe or severe halitosis; 2) sensitive cut-off point (score > 1) = very severe, severe, or moderate halitosis

Table 3 presents the distribution of the VSC and ICP scores compared to the OA at the sensitive cut-off point. Except for the specific VSC cut-off point (80 ppb), in the other cut-off points of VSC, we observed a predominance of true positive values (TP). In the distribution analysis of ICP scores compared to OA, false negative results were predominant.

**Table 3:** Distribution of volatile sulfur compounds (VSC) scores and Interview with a close person (ICP) compared to organoleptic assessment by a trained professional (OA) at the sensitive cut-off point (i.e. bad breath means very severe, severe, or moderate halitosis)

Halitosis		Organoleptic Judgment (OJ) – sensitive cut-off		Total
		Yes	No	
Test:	Yes	84	21 (49%)^1^	105
VSC	No	11 (12%)^2^	22	33
Cut off = 35ppm	**Total**	95	43	138
Test:	Yes	68	9 (21%)	77
VSC	No	27 (28%)	34	61
Cut off = 50ppm	**Total**	95	43	138
Test:	Yes	23	2 (5%)	25
VSC	No	72 (76%)	41	113
Cut off = 80ppb	**Total**	95	43	138
Test:	Yes	34	8 (26%)	42
ICP Cut off sensitive	No	40 (54%)	23	63
	**Total**	74	31	105
Test:	Yes	7	2 (6%)	9
ICP Cut off specific	No	67 (91%)	29	96
	**Total**	74	31	105

Percentage of ^1^false positives and ^2^false negatives.

Interview with a close person scores cut-off points: 1) specific cut-off point (score>2) = very severe or severe halitosis; 2) sensitive cut-off point (score>1) = very severe, severe, or moderate halitosis

Tables 4 , 5 , and 6 present the properties of the diagnostic tests (sensitivity and specificity), predictive values, and results of concordance tests.

**Table 4:** Accuracy of volatile sulfur compounds (VSC) compared to organoleptic assessment by a trained professional (OA) at the specific cut-off point

	OJ (cut-off: score >=3) vs. VSC (cut-off>=65ppb)	OJ (cut-off: score >=3) vs. VSC (cut-off >=80ppb)	OJ (cut-off: score >=3) vs. VSC (cut-off >=140ppb)
Agreement global	0,78	0,86	0,9
Kappa 95%CI	0.41 (0.26 to 0.57)	0.40 (0.27 to 0.53)	0.48 (0.25 to 0.71)
MacNemar X2	26.13 (p-v < 0.001)	3.20 (p-v < 0.07)	1.14 (p-v < 0.29)
Sensitivity 95%CI	0.94 (0.90 to 0.98)	0.65 (0.57 to 0.73)	0.47 (0.39 to 0.55)
Specificity 95%CI	0.76 (0.69 to 0.83)	0.88 (0.83 to 0.94)	0.96 (0.93 to 0.99)
PPV 95%CI	0.36 (0.28 to 0.44)	0.44 (0.36 to 0.52)	0.62 (0.53 to 0.70)
NPV 95%CI	0.99 (0.97 to 1.07)	0.95 (0.91 to 0.98)	0.93 (0.88 to 0.97)
OJ Prev (Gold Standard)	0.12 (0.07 to 0.18)		
VSC prev 95%CI	0.33 (0.25 to 0.40)	0.18 (0.12 to 0.25)	0.09 (0.05 to 0.14)

OA = Organoleptic assessment by a trained professional; PPV = Positive Predictive Value; NPV = Negative Predictive Value; Prev = Prevalence; ppb = parts per billion; p-v = p-value; CI = Confidence Interval

Organoleptic assessment scores cut-off points: 1) specific cut-off point (score > 2) = very severe or severe halitosis; 2) sensitive cut-off point (score > 1) = very severe, severe, or moderate halitosis

**Table 5:** Accuracy of volatile sulfur compounds (VSC) compared to organoleptic assessment by a trained professional (OA) at the sensitive cut-off point

	OJ (cut-off: score >=2) vs. VSC (cut-off >=35ppb)	OJ (cut-off: score >=2) vs. VSC (cut-off >=50ppb)	OJ (cut-off: score >=2) vs. VSC (cut-off >=80ppb)
Agreement global	0,77	0,74	0,46
Kappa 95%CI	0.42 (0.26 to 0.59)	0.45 (0.31 to 0.60)	0.14 (0.05 to 0.22)
Mac Nemar X^2^	3.13 (p-v < 0.08)	9.00 (p-v < 0.001)	62.22 (p-v < 0.001)
Sensitivity 95%CI	0.88 (0.83 to 0.94)	0.72 (0.64 to 0.79)	0.24 (0.17 to 0.31)
Specificity 95%CI	0.51 (0.43 to 0.60)	0.79 (0.72 to 0.86)	0.95 (0.92 to 0.99)
PPV 95%CI	0.80 (0.73 to 0.87)	0.88 (0.83 to 0.94)	0.92 (0.87 to 0.97)
NPV 95%CI	0.67 (0.59 to 75)	0.56 (0.47 to 0.64)	0.36 (0.28 to 0.44)
OJ prev (Gold Standard)	0.69 (0.61 to 0.77)		
VSC prevalence 95%CI	0.76 (0.69 to 0.83)	0.56 (0.47 to 0.64)	0.18 (0.12 to 0.25)

OA = Organoleptic assessment; PPV = Positive Predictive Value; NPV = Negative Predictive Value; Prev = Prevalence; ppb = parts per billion; p-v = p-value; CI = Confidence Interval

Organoleptic assessment scores cut-off points: 1) specific cut-off point (score > 2) = very severe or severe halitosis; 2) sensitive cut-off point (score > 1) = very severe, severe, or moderate halitosis

**Table 6:** Accuracy of the interview with a close person (ICP) compared to organoleptic assessment by a trained professional (OA)

	OJ (cut-off: score >=2) vs. ICP (cut-off: score >=2)	OJ (cut-off: score >=3) vs. ICP (cut-off: score >=3)
Agreement global	0,54	0,82
Kappa 95%CI	0.16 (0 to 0.30)	0.08 (-0.15 to 0.3)
MacNemar X2 95%CI	21.33 (p-v < 0.001)	1.32 (p-v < 0.25)
Sensitivity 95%CI	0.46 (0.36 to 0.55)	0.14 (0.08 to 0.21)
Specificity 95%CI	0.74 (0.66 to 0.83)	0.92 (0.87 to 0.97)
PPV 95%CI	0.81 (0.73 to 0.88)	0.22 (0.14 to 0.30)
NPV 95%CI	0.37 (0.27 to 0.46)	0.86 (0.81 to 0.94)
OJ prev (Gold Standard)	0.69 (0.61 to 0.77)	0.12 (0.07 to 0.18)
ICP 95%CI	0.35 (0.28 to 0.41)	0.09 (0.03 to 0.14)

OA = Organoleptic assessment; ICP = Interview with a close person, PPV = Positive Predictive Value; NPV = Negative Predictive Value; Prev = Prevalence; p-v = p-value; CI = Confidence Interval

Organoleptic assessment and interview with a close person scores cut-off points: 1) specific cut-off point (score>2) = very severe or severe halitosis; 2) sensitive cut-off point (score>1) = very severe, severe, or moderate halitosis

Table 4 describes VSC properties compared to the OA’s sensitive halitosis definition. Halitosis prevalence of was 12%, negative predictive values (~NPV) were high among all analyzed cutoff points (≥0.93), whereas positive predictive values (PPV) showed less satisfactory values (≤0.62). The global agreement was high, ranging from 0.78 to 0.90. The Kappa and McNemar concordance tests indicated that the most specific VSC cut-off (≥140 ppb) was the one that showed the greatest concordance with the OA. VSC ≥65 ppb showed high sensitivity (0.94) and moderate specificity (0.76). The VSC ≥140 ppb, on the other hand, showed high specificity (0.96) and low sensitivity (0.47).

Table 5 describes the properties of the VSC test compared to the OA’s sensitive halitosis definition. The prevalence of halitosis was 69%, PPV was high (≥0.80) in all analyzed cut-off points, whereas negative predictive values (NPV) showed less satisfactory values (≥0.67). The global agreement ranged from 0.46 to 0.77. The Kappa coefficient indicated greater agreement between the OA and VSC at the intermediate cut-off point (cut-off ≥50 ppb). VSC, at cut-off ≥80 ppb, showed high specificity (0.95), but low sensitivity (0.24).

Table 6 refers to the properties of the ICP, in which the sensitive and specific cut-off points of ICP and OA are compared to each other. A high NPV (0.86) is observed at the ICP specific cut-off point, and a high PPV at the ICP sensitive cut-off point (0.81). The global agreement was high only at the specific cut-off point (0.82). High specificity (0.92) is observed between the ICP and OA specific cut-offs. The sensitivity in both tests was less than 0.5.

## Discussion

Previous studies have estimated that the prevalence of halitosis varies between 2% and 49% in different populations^8 , 25^ . It is difficult to make comparisons between the estimates obtained in the various halitosis prevalence surveys since variations could be explained by the different methods and criteria used to define the presence of halitosis, in addition to the subjectivity and low reproducibility of organoleptic measurements.^26 , 27^

This study showed excellent information reproducibility from a close person to detect the presence of severe or very severe halitosis. No other studies were found with information on the data reproducibility from a close person on halitosis.

In this study, the information from a close person about the presence of severe or very severe halitosis was accurate, comparable with the organoleptic assessment by a trained professional. The accuracy of the close person was demonstrated by a high global agreement and similar prevalence, with largely overlapping 95% confidence intervals, compared to the organoleptic assessment by a trained professional. The prevalence of severe or very severe halitosis in this study as defined by a close person (12%, 95%CI: 7% to 18%) or by a trained professional (9%, 95%CI: 3% to 14%) was similar to the 15% prevalence of chronic halitosis found in a previous study that relied on the information from a close person.^7^

The VSC was the test that stood out the most, both to detect negative (specificity: 96%, cut-off 140 ppb) and positive cases of halitosis (sensitivity: 94% cut-off 65 ppb). Although the ICP was also accurate in capturing truly negative cases of severe or very severe halitosis (specificity: 92%), it presented the worst sensitivity (<50%). Previous studies found a sensitivity that varied from 52% to 90% and specificity from 45% to 90% for VSC.^6 , 28 , 29^

The diagnostic strategy aiming for maximum specificity of both VSC and ICP seems to be more appropriate when the main concern is to avoid a false-positive result. This situation can be interesting in cases that the patient, supposedly with bad breath, goes to a specialized gastroenterology service, assuming that the cause of bad breath is gastrointestinal. Considering that a positive test result may indicate the need for invasive and costly procedures, the inclusion of a halitosis test with high specificity in the care protocol, before decisions involving a higher level of complexity (such as digestive endoscopy) can avoid unnecessary costs and discomfort for the patient. One study found that about 57% of patients who sought treatment for halitosis had some gastrointestinal pathology, most of them in the stomach and related to *Helicobacter pylori* infection.^30^ Another study also detected the presence of *H. pylori* bacteria in 91% of patients with halitosis and only in 32% of patients without halitosis.^31^

On the other hand, the strategy aiming at greater sensitivity in halitosis measurements may fit in cases that require simple treatments that are easy to perform. This may be the case of a patient who seeks dental service with a complaint of halitosis. A test with high sensitivity, even though it generates a higher percentage of false positives, might be a more appropriate alternative since most cases of halitosis are solved with simple and low-complexity procedures, such as changing habits of oral hygiene, among others.^32 - 35^ It is noteworthy that about 90% of halitosis cases originate in the oral cavity, which provides a suitable environment for bacterial growth. These bacteria are mainly retained on the tongue and periodontium and can cause halitosis.^3^

In the present study, the area under the ROC curve ranged from 78 to 89%, but a previous study found an area under the ROC curve of 67% for VSC.^12^

The VSC test in the present study showed both high sensitivity (94%) at the 65ppb cut-off point and high specificity (96%) at the cut-off point of 140ppb. The Kappa agreement coefficient ranged from 0.40 to 0.48. Previous studies reported agreement between VSC and OA that varied around 0.60.^12 , 28 , 36^ The differences found between our study and previous studies could be explained by the different methods and criteria used to define the presence of halitosis and the types of coefficients used to compare the test and the gold standard.

The ICP presented low sensitivity but high specificity. A previous study that investigated if the patient had been warned about the presence of halitosis by a close person, found a higher sensitivity (82%) than our study^7^ . These different results could be due to different strategies for collecting information and the type of population studied.

OA and ICP may not detect the same aspect of halitosis. The OA is a point measure of halitosis status, therefore, it can either express an occasional moment of bad breath at the time of the exam, or it can characterize a chronic case of halitosis. The ICP, on the other hand, can be a potential instrument to detect chronic halitosis. Therefore, an argument could be made that, if the aim is to detect the presence of chronic halitosis, the ICP could become the gold-standard, with the OA as the alternative diagnostic test. The predictive values in the present study could be the exact measures of sensitivity and specificity if we reversed their roles, that is, if ICP became the gold-standard and the organoleptic assessment became the alternative test. Making this exchange, instead of finding 46% and 92% as maximum values of sensitivity and specificity, we would find 81% and 86% as maximum values of sensitivity and specificity, respectively, of the OA compared to the ICP as the gold-standard.

Although it was not the focus of this study, performing repeated measurements of oral odor over time to detect the presence of chronic halitosis could be a way of confirming its presence since, in this study, we only focused on the question “In general, how do you evaluate the breath odor of (the patient’s name)?” to characterize the presence of persistent halitosis. New investigations with this focus could contribute to elucidate the role of ICP in chronic halitosis detection.

Among the limitations of this study, we emphasize that, for logistic reasons, we had to work with samples of patients treated at a state university hospital and not with a random sample of the general population. However, even if there was difference between the halitosis profile of the participants and general population, it would not interfere with the main purpose of this study, which was to assess the accuracy of the VSC and the ICP, compared to the OA.

A second limitation is the lost 25% of the invited patients to participate in the test retest. What could explain this, in addition to the two-week interval between the first and second interviews, is the fact that the second interview was by telephone.

A third limitation is that all patients were fasting, which could have contributed to an increase in the prevalence of halitosis. However, it is unlikely that this influenced the accuracy of the halitosis measuring methods.

No information was collected on participants’ eating habits, medications, oral health, and oral hygiene habits. Although these factors may contribute to the presence of halitosis, we have no reason to believe that they interfered with the accuracy study.

Sensitivity, specificity, and predictive values are useful measures in the evaluation of diagnostic tests. However, clinical benefits, economic burdens, and advantages and disadvantages over other tests also need to be considered. Knowledge of the techniques for validating and interpreting diagnostic tests is, therefore, essential for health professionals so that they can guide their decisions about the real usefulness of tests on a scientific basis.

## Conclusion

The VSC presents high sensitivity at the cut-off point of >65 ppb and high specificity at the cut-off point of >140 ppb, however, the best test characteristics were detected at the cut-off point of > 80 ppb (Sensitivity: 0.65 and specificity 0.88). ICP had high specificity, but low sensitivity. The OA can express either occasional or chronic bad breath, whereas the ICP can be a potential instrument to detect chronic halitosis.
